# Urease Inhibitory Activities of some Commonly Consumed Herbal Medicines

**Published:** 2015

**Authors:** Shabnam Mahernia, Kowsar Bagherzadeh, Faraz Mojab, Massoud Amanlou

**Affiliations:** a *Department of Medicinal Chemistry, Faculty of Pharmacy and Medicinal Plants Research Center, Tehran University of Medical Sciences, Tehran, Iran.*; b *Department of Pharmacognosy, School of Pharmacy, Shahid Beheshti University of Medical Sciences, Tehran, Iran.*

**Keywords:** Urease, *Helicobacter pylori*, Medicinal plants, Inhibitor, Gastric diseases

## Abstract

Urease enzyme has a crucial role in the persistent habitation of *Helicobacter pylori* (*H. pylori*) that induces gastrointestinal diseases, in particular gastritis, duodenal, peptic ulcer, and gastric cancer. Plants have long been utilized as the biggest source of substances with medicinal properties from natural origin and therefore result in less toxicity and adverse side effects upon usage. 15 medicinal plant extracts were examined against Jack bean urease activity by Berthelot reaction. Each herb was extracted using 80% aqueous methanol. The more effective extracts were further tested and their IC_50_ values were determined. Three plant extracts including *Ginkgo biloba*, *Rhus coriaria,* and *Matricaria inodora *were found to be the most effective ones with IC_50_ values of 36.17, 80.29, and 100.6 μg/mL, respectively.

## Introduction

Urease, the enzyme responsible for the rapid hydrolysis of urea to ammonia, is a key enzyme benefiting bacteria *H. pylori *through making its persistence possible in the acidic environment of the stomach and as a result, cause gastrointestinal diseases, in particular gastritis, duodenal, peptic ulcer, and gastric cancer (1,2). It has already been proven that urease deficiency effectively risks the bacteria existence (3). Additionally, urease activity leads to other disease like urinary stones, pyelonephritis (4). Also limiting nitrification procedure via restricting urease hydrolysis activity is also of great importance in the field of agriculture to control nitrogen leaching, greenhouse gas escape and ammonia volatilization from soil (5).

Although comprehensive studies have been performed on urease inhibition mechanisms and inhibitors until today, only a few of them are promising. According to the literature, most of the prescribed medicines and antibiotics for the mentioned disease treatment, not only evince adverse effects but also the bacteria grow resistance against (6). Furthermore, application of some compounds and especially synthetic ones for controlling urease function has been banned due to their toxicity and low chemical and physical stability in the natural environment (5). Medicinal plants have long been applied as remedies to cure diseases which nowadays are known as viral infections. Composites from *Euphorbia decipiens* (7) and sulfated polysaccharide found in different types of brown seaweed (fucoid an compounds) had been previously reported, are examples of natural substances with urease inhibition activities (8). While plants can be considered as the largest source of substances with pharmacological properties, their significant biological characteristics have not been investigated thoroughly. As a result, it is obvious that looking for efficient composites with natural origins to be used individually and/or as lead ones to design and develop new drugs with higher efficiency, stability and less toxicity is an important issue need to be more attention to.

Considering the vital role of urease enzyme in the immune system of the bacteria *H. pylori,* this work has focused on monitoring and evaluating inhibition activity of 15 herbs for their possible inhibitory activity against Jack bean urease. Therefore, the extracts of some selected medicinal herbs commonly consumed in Iranian traditional medicine were prepared and their inhibition strength was examined upon Jack bean urease (9).

## Experimental

*Material*

All chemicals used were of analytical grade from Merck Co., Germany. Sodium nitroprusside and urease (EC 3.5.1.5) from Jack beans were purchase from sigma (St. Louis, MO, USA). Ultra-pure water (HPLC grade, Duksan, Korea) was used throughout the experiments. Potassium phosphate buffer (100 mM), pH=7.4, was prepared in distilled water. The studied plants were collected from Herbarium of School of Pharmacy, Tehran University of Medical Sciences. The absorbance spectra of the solutions were obtained employing Synergy H1 Hybrid multi-mode microplate reader.

*Plants extraction*

The studied medicinal plants (Table 1) were gathered from the Herbarium of Faculty of **Pharmacy**, Tehran University of Medical Sciences, air dried and then powdered. 0.5 g of the obtained powder was extracted in 10 mL, methanol:water (80:20; V/V) at room temperature (25 ± 1 ºC) for 24 hours, filtered and then dried under reduced pressure and finally freeze dried. The dry extracts were stored at -20 ºC till used (10).

*Determination of urease activity*

Initial urease inhibitory activity of all the obtained extracts was evaluated at the concentration of 1 mg/mL with the modified Berthelot spectrophotometric method at the absorbance of 625 nm. Finally, IC_50_ inhibitory activity of each extract was assessed. Also, inhibition activity of hydroxyurea was assayed as the standard compound which is already proved to have significant inhibitory characteristics for urease. 

The assay solution mixture consisted of urea (850 µL), the extract (in the range of 0 to 100 µL) and phosphate buffer (100 mM, pH 7.4) to reach the total value of 985 µL. The enzymatic reactions started with the addition of 15 µL of urease enzyme and measured via determining ammonia concentration after 60 minutes using 500 µL of solution A (contained 0.5 g phenol and 2.5 mg of sodium nitroprusside in 50 mL of distilled water) and 500 µL of solution B (contained of 250 mg sodium hydroxide and 820 µL of sodium hypochlorite 5% in 50 mL of distilled water) at the temperature of 37 °C for 30 minutes. Activity of uninhibited urease was chosen as the control activity of 100%.

*Data processing & IC*_50_ *Determination*

The following equation was employed to calculate the enzymatic reaction value:

***I*** *(%) = [1 –* *(T / C)]*_ *_ *100*

Where *I* (%) is the inhibition of the enzyme, T (test) is the absorbance of the tested sample (plant extract or positive control in the solvent) in the presence of enzyme, C (control) is the absorbance of the solvent in the presence of enzyme. Data are expressed as mean ± standard error (SD) and the results were taken from at least three times.

The concentration that induces an inhibition halfway between the minimum and maximum response of each compound (relative IC_50_) was determined monitoring the inhibition effect of various concentrations of compounds in the assay. The IC_50_ values were then calculated using GraphPad Prism 5 software (Table 1).

**Table 1:** Urease inhibitory activity of the studied plant extracts and the calculated IC_50_ values

**IC**_50_ ** (μg/mL)**	**Common name**	**Plant Family**	**Scientific name**	
**226.00**	Marshmallow	Malvaceae	*Althaea officinalis L* *.*	1
**537.80**	Toothpick	Apiaceae	*Ammi visnaga L.*	2
**935.10**	Sacred datura	Solanaceae	*Datura inoxia Miller*	3
**719.30**	Datura	Solanaceae	*Datura stramonium L.*	4
**131.60**	Purple coneflower	Asteraceae	*Echinacea purpurea L.*	5
**36.17**	Ginkgo	Ginkgoaceae	*Ginkgo biloba L.*	6
**100.60**	German chamomile	Asteraceae	*Matricaria inodora L* *.*	7
**80.29**	Sumach	Anacardiaceae	*Rhus coriaria L* *.*	8
**607.80**	Soapwort	Caryophyllaceae	*Saponaria officinalis L.*	9
**409.90**	Silver ragwort	Asteraceae	*Senecio cineraria DC.*	10
**140.50**	Milk thistle	Asteraceae	*Silybum marianum L.*	11
**307.20**	Spanish broom	Leguminosae	*Spartium junceum L.*	12
**161.70**	Spiraea	Rosaceae	*Spiraea crenata L.*	13
**341.80**	Common tansy	Asteraceae	*Tanacetum vulgare L.*	14
**264.50**	Common yew	Taxaceae	*Taxus baccata L.*	15
**37**			*Hydroxyurea*	16

## Results and Discussion

 Natural therapy has recently absorbed many attentions to itself. Although, herbs have always been applied for the treatment of a vast variety of diseases throughout the history, but the drawbacks of the synthetized medicines, especially the side effects coming along their consumption, has again arisen a significant interest among scientists to more precisely monitor and extract herbal active compounds pharmacological properties from these appropriate sources of active chemicals to be used as templates for designing and/or developing more effective compounds, preferably with less side effects (11).

Gastrointestinal disorders, particularly gastritis, duodenal, peptic ulcer, and gastric cancer are mainly caused as a result of *H*. *pylori *infection. This bacterium agitates human pathogenic state and causes diseases from which the most common ones are urinary stone formation, peptic ulcer, pyelonephritis, and hepatic coma. *H. pylori *habitance in the acidic medium of the stomach is highly dependence on the urease enzyme activity. A unique feature of *H. pylori* infection is its persistence as a result of urease enzyme buffering activity. The enzyme changes the stomach medium to an endurable environment for the bacteria via neutralizing gastric acid through hydrolysis of urea to form carbon dioxide (CO_2_) and ammonia (NH_3_) (12).

Herein, urease enzyme inhibitory activity of 15 natural extracts were evaluated among which 3 extracts were elucidated as the most potent ones including *Rhus coriaria, Ginkgo biloba*, and *Matricaria inodora*. As it is presented in Table 1,* G. biloba *with IC_50_ value of 36.17 μg/mL (even lower than that of hydroxyurea, the positive control, with the IC_50_= 37 μg/mL) is the most effective compound followed by *R. coriaria*, and *M. inodora* with IC_50_ values of 80.29 and 100.6 μg/mL, respectively.

**Figure 1:**
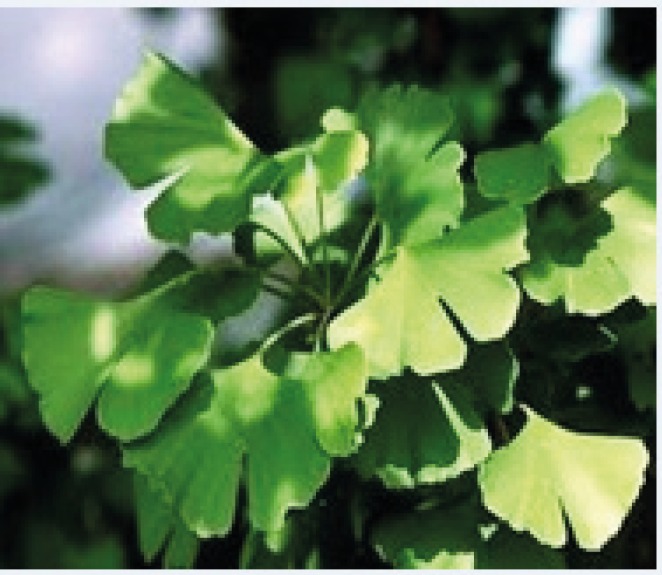
*Ginkgo biloba* leaf (14).

*Ginkgo biloba *(Figure 1) has shown to contain different biflavones with antilipoperoxidant, anti-necrotic and radical-scavenging properties together with diverse flavones that have proven to show anti-influenza A, anti-herpes simplex virus 1 and 2 (HSV-1 and HSV-2) activities (13, 14).

**Figure 2:**
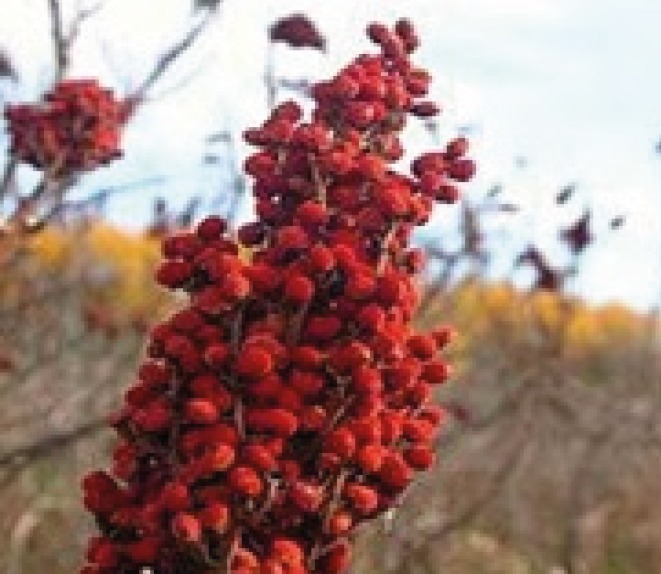
*Rhus coriaria* fruit (18).

*Rhus coriaria *(Figure 2) has been applied as a medicinal plant since prehistoric times. Sumacis reported to have hydrolysable tannins, gallo-tannins, volatile oil, flavonoids, anthocyanin, gallic acid, and flavones, such asmyricetin, quercetin and kaempferol. According to the literature survey, Sumac has been documented to reveal antibacterial, hepatoprotective, antifungal, antioxidant anti-inflammatory/chondroprotective, and many other medicinal characteristics (15). Also anti-helicobacter pylori effect of it has been previously reported (16,17). The plant was traditionally used to treat diarrhea, dysentery, leucorrhea, and stomach tonic. Other studies also mentioned *R. coriaria *application as an antimicrobial agent (13).

**Figure 3:**
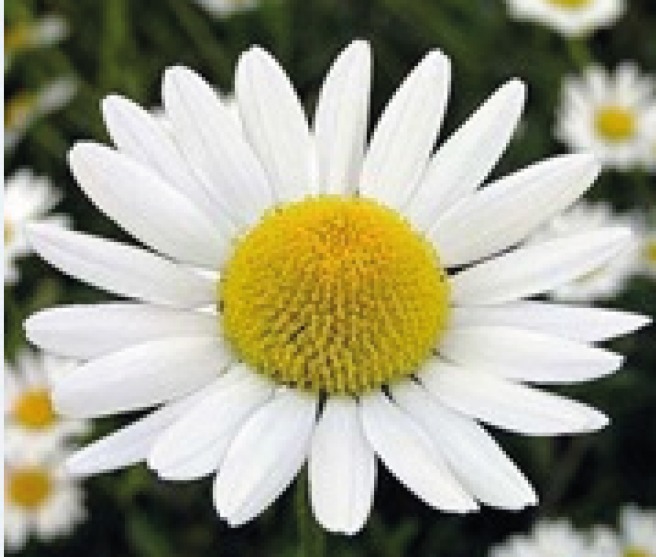
*Matricaria inodora* flowering stage (19).

*Matricaria* *inodora *(Figure 3) includes aromatic substances called carminatives that help the eructation reflux and as a result act as a gastrointestinal tract pain reliever. It has also been reported to contain flavonoids constitutes and as a result can act as anti-inflammatory operator. The plant has long been used to relieve indigestion, promote appetite, and also for the treatment of stomach ulcers to help prevent food poisoning and treat gastric ulcer, swollen liver and spleen (13).

The results from this study along with the ones from the literature (6,10,14-17,20,21) confirm some of the traditional applications of the above mentioned medicinal plants (Table 1) as gastric tonics, anti-ulcers, anti-bacterial, indigestion reliever and many others (14-17). These activities may be mediated by direct inhibition of bacterial urease such as *H. pylori* urease and their subsequent anti-*H. pylori *activity or indirect action as an anti-oxidant, anti-inflammatory agents or pH-mediator compounds through their active chemical constituents (14-17). The obtained IC_50_ values for the three most potent extracts were lower than those of others with urease inhibitory activity reported recently (6,10,20,21). This finding promotes the capability of these plants (*M. inodora, G. biloba* & *R. coriaria*) to be used as herbal remedies to manage gastrointestinal tracts ailment alone or along with other official anti-*H. pylori* drugs.

## References

[1] Devesa SS, Blot WJ, Fraumeni JF (1998). Changing patterns in the incidence of esophageal and gastric carcinoma in the United State. J. Cancer.

[2] Howson CP, Hiyama T, Wynder EL (1986). The decline of gastric cancer: epidemiology of unplanned triumph. J. Epidemiol. Rev.

[3] Michetti K (1998). Pathogenesis of Helicobacter pylori infection. Curr. Opin. Gastroenterol.

[4] Upadhyay LS (2012). Urease inhibitors: A review. Indian J. Biotech.

[5] Edmeades DC (2004). Nitrification and urease inhibitors.

[6] Golbabaei S, Bazl R, Golestanian S, Nabati F, Bahrampour Omrany Z, Yousefi B, Hajiaghaee R, Rezazadeh S, Amanlou M (2013). Urease inhibitory activities of β-boswellic acid derivatives. Daru.

[7] Ahmad V, Hussain J, Hussain H, Jassbi AR, Ullah F, Lodhi MA, Yasin A, Iqbal M (2003). First natural urease inhibitor from Euphorbia decipiens. Chem. Pharm. Bull.

[8] Limuro M, Wakabayashi K (2003). Preventive effects of Cladosiphonfucoidan against Helicobacter pylori infection in mongolian gerbils. Helicobacter.

[9] Benini S, Rypniewski WR, Wilson KS, Miletti S, Ciurli S, Mangani S (1999). The complex of Bacillus pasteurii urease with acetohydroxamate anion from X-ray data at 1.55 Å resolution. J. Biol. Inorg. Chem.

[10] Nabati F, Mojab F, Habibi-Rezaei M, Bagherzadeh K, Amanlou M, Yousefi B (2012). Large scale screening of commonly used Iranian traditional medicinal plants against urease activity. Daru.

[11] Heinrich M, Barnes J, Gibbons S, Williamson EM (2004). Fundamentals of Pharmacognosy & Phytotherapy.

[12] Kuwahara H, Miyamoto T, Kubota T, Sawa T, Okamoto S, Maeda H (2000). Helicobacter pylori urease suppresses bactericidal activity of peroxynitrite via carbon dioxide production. Infect. Immun.

[13] Evans WC (2009). Pharmacognosy.

[14] Introduction to the Ginkgoales. http://www.ucmp.berkeley.edu/seedplants/ginkgoales/ginkgo.html.

[15] Shabbir A (2012). Rhus coriaria Linn, A plant of medicinal, nutritional and industrial importance: a review. J. Anim. Plant. Sci.

[16] Fazeli MR, Amin G, Attari MMA, Ashtiani H, Jamalifar H, Samadi N (2007). Antimicrobial activities of Iranian sumac and avishan-e-shirazi (Zataria multiflora) against some food-borne bacteria. Food Control.

[17] Kossah R, Zhang H, Chen W (2011). Antimicrobial and antioxidant activities of Chinese sumac (Rhus typhina L ) fruit extract.. Food Control.

[18] Wikipedia contributors Sumac Wikipedia, The free Encyclopedia. http://en.wikipedia.org/wiki/Sumac.

[19] Wikipedia contributors Matricaria Wikipedia, The Free Encyclopedia. http://en.wikipedia.org/wiki/Matricaria.

[20] Biglar M, Soltani K, Nabati F, Bazl R, Mojab F, Amanlou M (2012). A preliminary investigation of the Jack-bean urease inhibition by randomly selected traditionally used herbal medicine. Iran. J. Pharm. Res.

[21] Biglar M, Sufi H, Bagherzadeh K, Amanlou M, Mojab F (2014). Screening of 20 Commonly Used Iranian Traditional Medicinal Plants Against Urease. Iran. J. Pharm. Res.

